# Little Difference in Milk Fatty Acid and Terpene Composition Among Three Contrasting Dairy Breeds When Grazing a Biodiverse Mountain Pasture

**DOI:** 10.3389/fvets.2020.612504

**Published:** 2021-01-22

**Authors:** Madeline Koczura, Bruno Martin, Marilena Musci, Martina Di Massimo, Matthieu Bouchon, Germano Turille, Michael Kreuzer, Joel Berard, Mauro Coppa

**Affiliations:** ^1^Université Clermont Auvergne, INRAE, VetAgro Sup, UMR1213 Herbivores, Saint-Genès-Champanelle, France; ^2^Eidgenössische Technische Hochschule Zürich, Institute of Agricultural Sciences, Universitaetstrasse, Zurich, Switzerland; ^3^Department of Food and Drug, University of Parma, Parma, Italy; ^4^Herbipôle, INRAE, Saint-Genès-Champanelle, France; ^5^Department of zootechnics, Institut Agricole Régional, Aosta, Italy; ^6^Eidgenössische Technische Hochschule Zürich, AgroVet-Strickhof, Lindau, Switzerland; ^7^Animal Production Systems and Animal Health, Agroscope, Zurich, Switzerland; ^8^Independent Researcher at Université Clermont Auvergne, INRAE, VetAgro Sup, UMR1213 Herbivores, Saint-Genès-Champanelle, France

**Keywords:** grazing behavior, simulated bites, late-lactating, Holstein, Montbéliarde, Valdostana Red Pied, α-linolenic acid (C18:3 n-3)

## Abstract

In the mountains, autochthonous and robust breeds are often used to valorize biodiverse grasslands. Along with their lower nutrient requirements, compared to specialized dairy breeds, they are expected to be better adapted to complex environments and valorize grasslands into dairy products of high quality. Therefore, the aim of the present study was to investigate the grazing selection of three contrasting dairy breeds on a biodiverse mountain pasture, and its consequences on milk fatty acid (FA) profile and prevalence of individual terpenes. A dual-purpose breed from the Italian Alps, the Valdostana Red Pied (Va), was compared to Montbéliardes (Mo), more specialized in milk production, and the highly specialized Holsteins (Ho). Diet selection was measured by scan-sampling, calculating selectivity indexes, and collecting simulated bites during two consecutive days in June (end of first grazing cycle) and July (second grazing cycle). Milk samples were collected at each milking during these experimental periods. Yield of milk and its fat and protein contents were measured. Milk FA and terpenes were analyzed by gas chromatographic methods. We tested the effects of breed, period and their interaction in a repeated mixed model, and calculated Pearson's correlations between behavioral data and milk FA as well as terpenes. The Va grazed less mature vegetation than Ho, but this difference was not sufficient to lead to a major breed effect on milk FA profile and prevalence of terpenes. However, the proportion of α-linolenic acid (C18:3 n-3) was always higher in the milk fat of Va than Ho (Mo were intermediary), but this without any correlation to grazing selection. This could be a consequence from a different metabolism concerning ruminal biohydrogenation, but must be further investigated. Finally, we confirmed previous studies that highlighted a link between milk quality and cows' grazing behavior, but here without differences among breeds. All cows adapted their behavior to the herbage evolution during the season, leading to higher proportions of unsaturated FA in July than June milks. Our study suggests that under mountain grazing conditions (biodiverse pasture and cows in late lactation), milk quality depends more on herbage composition than on cow breed.

## Introduction

Politics and consumers are increasingly concerned about the impact of livestock systems on environment and animal welfare, which triggers intensive research activities. Livestock systems need to be multi-functional. This means that they have to perform well in ecology, animal welfare and economics while producing healthy and quality food. The valorization of biodiverse pastures by ruminants is one of the key tools toward establishing this kind of livestock systems, characterized by higher resilience and fewer inputs than common systems (1). In mountain grazing dairy systems, autochthonous or robust cow breeds are commonly used to valorize these grasslands (2). Robustness is defined as an ability to adapt and carry on casual activities while facing environmental constraints (3). In the mountains, robust breeds with lower nutrient requirements, compared to specialized breeds, are expected to develop a natural resilience to their local environment and might better adapt to utilize the local grass resource, typically characterized by low nutritional value, and to valorize these resources by generating high-quality dairy products.

The ability to efficiently exploit local natural grasslands depends on grazing behavior, which can in turn influence the quality and specificity of dairy products (4). Plant secondary compounds (PSC) play a specific role in this context, such as phenols and terpenes, which are present in greater quantity in upland biodiverse grasslands (5–7). Besides species diversity, also vegetation stage and environmental conditions are involved in influencing the synthesis of terpenoids by the plants. For instance, Fraisse et al. (8) identified 170 different compounds on the same pasture, of which only 30 were common to all vegetation species and growth stages. These PSC may be transferred to the milk (9). Accordingly, milk from pastures rich in dicotyledons contains a greater quantity and wider diversity of terpenes than milk from pastures consisting mainly of grass species (10–12). Some PSC are also active in inhibiting the final reduction step of the biohydrogenation of fatty acids (FA) in the rumen (13–15). Therefore, when cows are grazing on biodiverse mountain pastures, their milk is richer in monounsaturated FA (MUFA), polyunsaturated FA (PUFA) and, especially, in conjugated linoleic acids (CLA) and n-3 FA (16–18). Terpenes and FA in milk are of interest for the sensory properties of milk and other quality attributes of the processed cheese (19).

Dairy cattle grazing on biodiverse pastures express preference or aversion for some specific plants that can vary according to their nutrient requirements and experience. Therefore, the actual diet ingested by the animals might differ from the average vegetation available on the pasture (20, 21), and this according to individual or breed-specific behavior (22, 23). Consequently, FA and terpene composition of the milk might vary among animals grazing the same biodiverse grassland. We reported a first part of the present study in Koczura et al. (24), showing that specialized dairy breeds with high nutrient requirements like Holstein cows (Ho) selected more grasses than dual-purpose breeds like Valdostana Red Pied cows (Va), an autochthonous Italian alpine breed with low requirements, when grazing on heterogenous and biodiverse pastures. The Va were generally less selective toward forbs and mature vegetation. Hence, the aim of the present study was to (i) deepen the analysis of grazing behavior of the three contrasting breeds by quantifying their diet selection on the biodiverse pasture and (ii) further investigate the consequences of this behavior on FA profile and prevalence of terpenes in the milk. We hypothesized that, as a result of our behavioral observations (24), the ingested diet of autochthonous Va cows would be more diverse than that of highly specialized breeds like Ho. Diet of Montbéliarde cows (Mo), which are less specialized for high milk production than Ho and supposedly better adapted to grazing on upland pastures, would be intermediate. We expected that the milk of the individual cows and breeds selecting less grasses and more forbs would have a higher prevalence of MUFA, PUFA, n-3 FA and terpenes.

## Materials and Methods

### Animals and Experimental Pasture

The present experiment was carried out in 2017 at Marcenat, INRAE's experimental farm. Other aspects and details of the experiment were described in Koczura et al. (24), in which data obtained in the beginning of June 2017 had also been included. Twelve late-lactating dairy cows (four Ho, four Mo, and four Va) grazing on a biodiverse pasture since the beginning of June were monitored during two consecutive days in the end of June (end of first grazing cycle) and then in July (beginning of the second grazing cycle). Briefly, cows grazed (extensive continuous grazing conditions) a natural and highly biodiverse pasture (65 species; 48% of grasses, 13% of legumes, and 39% of forbs on ground cover), dominated by *Festuca* gr. *rubra* (18%), and *Agrostis capillaris* (15%). Before the start of the experiment, the botanical composition (% of ground cover) was determined using the vertical point-quadrat method (25) (Table 1). The characteristics of the herbage on the experimental plots is described in Table 2. Cows did not receive any supplementation with concentrate, and had free access to water and mineral supplements. The individual cow's potential intake capacity, calculated according to INRA (26), was used as an estimation of herbage intake.

**Table 1:** Botanical composition of the experimental plot.

**Species^a^**	**Specific contribution (% of ground cover)**
*Festuca* gr. *rubra*	18.4
*Agrostis capillaris*	15.3
*Trifolium repens*	7.4
*Achillea* gr. *millefolium*	5.3
*Anthoxanthum odoratum*	4.2
*Thymus* gr. *serpyllum*	3.4
*Avenula pubescens*	3.3
*Trifolium pratense*	2.7
*Dactylis glomerata*	2.5
*Veronica arvensis*	2.5
*Plantago lanceolata*	2.4
*Luzula* gr. *campestris*	1.9
*Helianthemum nummularium*	1.8
*Lathyrus pratensis*	1.8
*Cynosurus cristatus*	1.7
*Galium verum*	1.6
*Carex sempervirens*	1.6
*Viola tricolor*	1.5
*Festuca* gr. *ovina*	1.4
*Stellaria graminea*	1.3
*Cerastium arvense*	1.3
*Ranunculus* gr. *montanus*	1.3
*Lotus corniculatus*	1.2
*Cerastium holosteoides*	1.2
*Daucus carota*	1.2
*Rumex acetosella*	1.2
*Chamaespartium sagittale*	1.1
*Potentilla erecta*	1.1
*Cirsium eriophorum*	1.0

a *Species with specific contribution <1%, in decreasing specific contribution order: Ajuga reptans, Meum athamanticum, Poa pratensis, Silene vulgaris, Hieracium gr. pilosella, Saxifraga granulate, Alchemilla gr. vulgaris, Stachys gr. officinalis, Hypericum hirsutum, Leucanthemum vulgare, Deschampsia flexuosa, Knautia arvensis, Crepis capillris, Dianthus deltoids, Ranunculus bulbosus, Tragopogon pratensis, Dianthus sylvestris, Gentiana lutea, Trisetum flavescens, Ranunculus repens, Rumex longifolius, Sanguisorba minor, Briza media, Poa chaixii, Centaurea gr. jacea, Pimpinella major, Veronica chamaedrys, Poa trivialis, Vicia cracca, Galium album, Spergula arvensis, Cytisus scoparius, Conopodium majus, Bromus erectus*.

**Table 2:** Characterization of vegetation offered on the experimental plots.

**Item**	**Period**		**SEM**
	**June**	**July**	
**Proportion of vegetation type (%)**			
Grasses	60.0	47.1	3.64
Legumes	5.6	6.8	1.22
Forbs	34.5	46.1	4.28
Short vegetation	42.0	54.1	4.05
Tall vegetation	45.9	26.2	3.56
Mature vegetation	12.1	19.7	2.50
**Nutritional value (g/kg DM)**			
Crude protein	8.3	9.1	0.24
Neutral detergent fiber	62.1	64.6	1.11
Acid detergent fiber	33.9	34.0	0.42
Digestibility of organic matter (g/kg)	45.6	42.3	0.94

### Grazing Behavior: Selectivity Index and Composition of Simulated Bites

During the two-day experimental periods, behavioral observations were performed each day by scan-sampling of the cows' bites at 5-min intervals. From these data, Jacob's index of selectivity (IS) was calculated as described in detail by Koczura et al. (24). These indices range from −1 (aversion) to +1 (preference). Additionally, on the same observation days, simulated bites were collected, according to the procedure described by Coppa et al. (27). Individual simulated bites were sampled several times for each cow during the days of observation, and immediately stored at 4°C. The different simulated bite samples were pooled to constitute one sample per period per cow, in order to be representative for the grazed herbage. From this herbage sample, two sub-samples were created: one of them was oven-dried at 60°C for 72 h and analyzed for crude protein (CP) (28), neutral detergent fiber (NDF) and acid detergent fiber (ADF) contents (29), as well as solubility in pepsin and cellulase (30) as an estimate of the organic matter digestibility (OMD). The second sub-sample was sorted into green and dry vegetation. Then, the green herbage was sorted into the three main botanical groups: grasses, legumes and forbs. All fractions were separately oven-dried (in the same conditions as these applied to the first sub-sample) and weighed. The proportion of each fraction to total dry matter (DM) was calculated and was referred to potential intake capacity to estimate the relative intake.

### Milk Sampling

In each experimental period, the individual milk yield of the cows was monitored at each milking during the two consecutive observation days, and 100-mL milk samples were collected. A 30-mL sub-sample was preserved with bronopol-B2, stored at 4°C and analyzed for fat and protein contents by Fourier transformed infrared spectroscopy [MilkoScan 4000, Foss System, Hillerød, Denmark, (31)] following the International Dairy Federation (32). Another sub-sample was stored at −20°C until further analyses.

### Milk Fatty Acids Analysis

A 3-mL milk sample was stored at −20°C before lyophilization (Thermovac TM-20, Froilabo, Ozoir-La-Ferrière, France) for FA analysis, performed as described by Ferlay et al. (33). Lipids were directly methylated using 2 mL of 0.5 M Sodium methoxide plus 1 mL of hexane at 50°C for 5 min, followed by cooling with the addition of 75μl of 12M HCl at room temperature for 10 min. The FA methyl esters were recovered in 3 mL hexane and washed with 3 mL water. Samples were injected by auto-sampler into a TraceGC 2000 series gas chromatograph equipped with a flame ionization detector (Thermo Finnigan, Les Ulis, France). Methyl esters from all the samples were separated on a 100 m 30.25 mm i.d. fused-silica capillary column (CP-Sil 88, Chrompack, Middelburg, The Netherlands). The injector temperature was maintained at 250°C and the detector temperature at 255°C. The initial oven temperature was held at 70°C for 1 min, increased by 5°C/min to 100°C (held for 2 min), and then increased by 10°C/min to 175°C (held for 40 min), and 5°C/min to a final temperature of 225°C (held for 15 min). The carrier gas was hydrogen. Identification of trans isomers of 18:1, non-conjugated 18:2, and CLA isomers was as described in Loor et al. (34). A reference standard butter (CRM 164, Commission of the European Communities, Community Bureau of Reference, Brussels, Belgium) was used to estimate correction factors for short-chain FA (C4:0 to C10:0). The *de novo* synthesized FA were defined as the sum of individual FA synthesized in the mammary gland.

### Milk Terpene Analysis

A balanced sample of milk from the four 50-mL samples from morning and evening milking that had been stored in 200-mL glass bottles at −20°C was thawed at ambient temperature for 6 h. The supernatant was then collected and centrifuged at 20,000 rpm and 25°C for 2 h. The anhydrous fat of these samples was collected and stored in 2 mL glass vials until further analysis for terpene composition. Then, headspace-solid phase microextraction (HS-SPME) was carried out using a 50/30 μm divinylbenzene-carboxen-polydimethylsiloxane (DVB/Carboxen/PDMS) fiber (Supelco, Bellefonte, PA, USA). The extraction conditions were the following: equilibration temperature: 60°C for 15 min; extraction temperature: 60°C for 60 min. The fiber was exposed into a gas chromatograph-mass spectrometer (GC-MS) injector for 2 min at 230°C, to desorb the terpenes. Analyses were performed on a Thermo Scientific Trace 1,300 gas chromatograph coupled to a Thermo Scientific ISQ single quadrupole mass spectrometer (both Thermo Fisher, Waltham, Massachusetts), equipped with an electronic impact source. All samples were injected in splitless mode, maintaining the valve closed for 3 min. The carrier gas was helium, with a total flow of 1 mL/min. The separation was performed on a SUPELCOWAX 10 capillary column (Supelco, Bellefonte, PA, USA, 30 m × 0.25 mm × 0.25 μm), using the following temperature program: starting at 35°C for 8 min; increasing by 4°C/min up to 60°C; increasing by 20°C/min up to 200°C; maintaining this final temperature for 10 min. The transfer line temperature was 230°C. The signal acquisition mode was SIM (93, 121, 136 m/z for the first 20 min, from 20 to 20,49 min were 93, 105, 133, 164, 204 m/z; from 20,50 min to the end were 93, 121, 147, 204 m/z). Terpenes identification was performed by comparing their retention times with those of pure standard compounds injected under the same chromatographic conditions, and comparing ratios between selected ions intensity with those of pure compounds.

### Statistical Analysis

Statistical analysis was performed with SAS software (version 8.6, SAS Institute Inc., Cary, NC). A repeated mixed model was applied, in which breed (Ho, Mo, Va), period (June, July) and their interaction were included as fixed factors. The repeated factor was the period, with the individual cow as subject. Results are presented as Least Square means and standard errors of the mean (SEM). In addition, Pearson's correlation coefficients between the variables describing grazing behavior and those of milk composition were calculated in order to be able to distinguish among individual cows.

## Results

### Grazing Behavior: Selectivity Index and Composition of the Simulated Bites

Only a few differences were observed among breeds in terms of grazing behavior (Table 3). Based on the IS, both Ho and Mo avoided mature vegetation whereas Va were indifferent to it. In June, Ho tended to express a stronger aversion to legumes than the other breeds (IS Ho = −0.78, IS Mo = −0.38, and IS Va = −0.25, *p* = 0.054; data not shown in table). No changes in the botanical and nutritional composition of the simulated bites was observed, except for a tendency of Mo to select herbage with a higher ADF content in June (+23 and +22 compared to that selected by Ho and Va cows, respectively, *p* = 0.093; data not shown in table). These tendencies observed in June among breeds were not observed in July. The daily potential intake capacity of Va was 4.2 kg of DM lower than those of Ho and Mo, and, as a consequence, their daily estimated grasses intake was lower (3.6 kg of DM). No difference among breeds were observed for legumes and forbs intake. The intake of dry material was also estimated higher for Ho compared to Mo and Va (+2.0 kg of DM). Regardless of breed, grazing behavior of all cows changed between the two experimental periods. In July, the IS for forbs and mature vegetation decreased by 0.31 and 0.62, respectively, whereas it increased by 0.17 and 0.25 for legumes and short vegetative herbage. The proportion of the different vegetation types in the selected bites did not differ between June and July, except for a tendency for increased proportion of dry vegetation (+12.5%, *p* = 0.059). Overall, the selected bites had a higher crude protein content (+38 g/kg DM) and a lower ADF content (−24 g/kg DM) in July compared to June. The estimated OMD tended to be higher by 2.9 g/kg DM in July than in June (*p* = 0.086). The proportion of legumes in the selected bites was negatively correlated to ADF and NDF contents (−0.609^**^ and −0.612^**^, respectively), and positively correlated to the crude protein content (0.673^**^) and estimated OMD (0.591^**^). The proportion of forbs in the simulated bites was negatively correlated to the NDF content (−0.462^*^). The proportion of dry material was negatively correlated to the ADF content (−0.432^*^).

**Table 3:** Indices of selectivity, characterization of vegetation eaten (simulated bites) by the cows, and performance distinguished by breed and period.

**Item**	**Breed**			**Period**			**Significance**		
	**Ho**	**Mo**	**Va**	**June**	**July**	**SEM**	**Breed**	**Period**	**Breed × period**
**Jacob's index of selectivity (−1 ≤ IS ≤ 1)**									
Grasses	0.45	0.41	0.29	0.41	0.36	0.030	ns	ns	ns
Legumes	−0.56	−0.36	−0.23	−0.47	−0.30	0.055	^†^	^*^	^†^
Forbs	−0.26	−0.27	0–.18	−0.08	−0.39	0.055	ns	^*^	ns
Short vegetation	0.39	0.47	0.28	0.26	0.51	0.046	ns	^*^	ns
Tall vegetation	−0.23	−0.28	−0.21	−0.27	−0.21	0.045	ns	ns	ns
Mature vegetation	−0.60^a^	−0.69^a^	−0.24^b^	−0.20	−0.82	0.093	^*^	^***^	^**^
**Proportion of vegetation type in the simulated bites (% of DM)**									
**Green vegetation**									
Grasses	86.6	81.2	84.4	84.1	84.0	4.05	ns	ns	ns
Legumes	1.2	2.2	1.3	0.5	2.6	0.75	ns	^*^	ns
Forbs	12.2	16.7	14.3	15.3	13.4	3.7	ns	ns	ns
Dry material	22.5	20.0	21.5	15.1	27.6	2.62	ns	^†^	ns
**Potential intake capacity and estimated intake of vegetation type (kg of DM)**									
Total potential intake capacity	22.7^a^	22.1^a^	18.2^b^	20.9	21.1	0.65	^**^	ns	ns
**Green vegetation**									
Grasses	15.7^a^	15.8^a^	12.1^b^	16.2	12.9	1.01	^*^	^**^	ns
Legumes	0.3	0.2	0.1	0.1	0.3	0.07	ns	ns	ns
Forbs	1.8	1.6	1.5	2.0	1.2	0.33	ns	^*^	ns
Dry material	4.3^a^	2.4^b^	2.1^b^	2.6	3.3	0.39	^**^	ns	^†^
**Nutritional value of the diet eaten by the cows (simulated bites; g/kg DM)**									
Crude protein	110	102	111	87	125	5.2	ns	^**^	ns
Neutral detergent fiber	658	653	642	657	645	4.5	ns	ns	ns
Acid detergent fiber	331	339	329	345	321	4.1	ns	^**^	^†^
Digestibility of organic matter (g/kg)	461	456	475	449	478	8.4	ns	^†^	ns
**Milk yield and composition**									
Milk yield (kg/day)	12.1	11.4	9.2	12.2	9.6	0.61	ns	^*^	ns
Protein content (g/kg)	30.6	33.0	30.6	31.6	31.2	0.74	ns	ns	ns
Fat content (g/kg)	39.5^b^	45.4^a^	36.2^b^	39.6	41.2	1.13	^**^	ns	ns
logSCC	2.4	3.2	2.2	2.2	2.4	0.07	ns	^*^	^*^
Protein yield (g/day)	363^a^	372^a^	278^b^	377	298	17.3	^†^	^**^	ns
Fat yield (g/day)	471^a^	510^a^	334^b^	484	393	25.4	^*^	^*^	ns

Significance levels:

*** p ≤ 0.001,

** p ≤ 0.01,

* p ≤ 0.05,

† *p ≤ 0.10, ns p > 0.10*.

a, b *Least square means with different superscript letters significantly differ at p < 0.05*.

*Ho, Holstein; Mo, Montbéliarde; SCC, somatic cell count; Va, Valdostana Red Pied*.

### Milk Yield and Gross Composition, and Their Relation With Grazing Behavior

Milk yield and protein content did not significantly differ among breeds. In Mo, the milk fat content was higher than in Ho and Va, by 5.9 and 9.2 g/kg, respectively. The daily milk fat yield was similar between Ho and Mo and lower by 126 g/day in average for Va. There was a similar tendency for the daily protein yield (−89 g/day in average for Va compared to both other breeds, *p* = 0.077). In June, the SCC of Mo's milk was lower than that of Ho and Va by 0.5 and 0.3 log units, respectively. Regardless of breed, the milk yield decreased by 2.6 kg from June to July. Concomitantly, the daily fat and protein yields decreased by 79 and 91 g/day, respectively. Milk yield and composition were not correlated to the proportions of the different botanical groups in the bites of the cows, but milk yield was positively correlated to the estimated grasses intake (0.594^**^), as well as milk fat and protein yields (0.620^**^ and 0.575^**^, respectively). However, both yields were also positively correlated to the total potential intake capacity (0.508^*^ and 0.575^**^, respectively), as well as milk fat content (0.406^*^). Concerning herbage composition, fat and protein yield were positively correlated to the ADF content (0.466^*^ and 0.497^*^, respectively). The SCC (log unit) of the milk was negatively correlated to the ADF and NDF contents of the herbage (−0.564^**^ and −0.457^*^, respectively).

### Milk Fatty Acid Profile and Its Relation With Grazing Behavior

The milk fat of Va tended to have a higher content of C18:2 n-6 (+0.11 g/100 g of total FA, *p* = 0.070) and had a higher content of C18:3 n-3 (+0.12 g/100 g of total FA) than the milk fat of both other breeds (Table 4). Milk fat of Mo tended to have the lowest content of total n-6 FA (−0.14 g/100 g of total FA in average, *p* = 0.097), and the n-6 to n-3 and C18:2n-6 to C18:3n-3 ratios were 10% higher in milk fat of Ho than that of the other breeds. In June, the milk fat of Ho tended to have a higher content of C16:0 (*p* = 0.071) than that of Mo (+0.30 g/100 g of total FA), and then Va (+1.5 g/100 g of total FA), but this was no longer the case in July (data not shown in table). Most of the changes in milk FA profile occurred between June and July. In June, the milk fat of all cows had a higher content of saturated FA (total SFA +2.9 g/100 g of total FA) than in July. More specifically, the content of C6:0, C8:0, C10:0, C12:0, C14:0, C15:0, and C16:0 was higher. On the contrary, the content of mono-unsaturated FA was higher in milk fat in July (+2.4 g/100 g of total FA), in particular that of C18:1c9 (+1.97 g/100 g of total FA), as well as the sum of all C18:1 *cis* isomers (+2.1 g/100 g of total FA). The content of poly-unsaturated FA in milk fat was also higher in July (+0.41 g/100 g of total FA), in particular that of n-3 FA (+0.09 g/100 g of total FA). Accordingly, the ratio n-6 to n-3 decreased in July. There was a decrease in the proportion of even-chained FA in July (−2.94 g/kg), whilst that of the odd-chained FA increased (+0.26 g/kg). The C14:1c9 to C14:0 and C18:1c9 to C16:0 ratios increased by 0.02 and 0.17 from June to July, respectively. *De novo* synthesized FA decreased in July, by 1.79 g/kg. Overall, milk FA proportions had little correlation with the proportions of the botanical groups in the diet of the cows. The proportion of grasses in the simulated bites was correlated only to the proportion of one FA, C18:0 (0.444^*^). The estimated grasses intake was also negatively correlated to C18:3n-3 (−0.482^*^) and n-3 FA (−0.412^*^) concentration in milk. In the same way, legume proportion was only correlated to the proportion of C18:3 n-6 (0.408^*^). Forb proportion was positively correlated to proportions of C12:0 and C14:0 (0.419^*^ and 0.423^*^, respectively), and negatively correlated to proportions of C14:1t9 and C18:0 (−0.444^*^ and −0.470^*^, respectively). The proportion of dry material in the bites was positively correlated to proportions of C18:1c9 (0.487^*^), MUFA (0.552^**^), sum of C18:1 *cis* isomers (0.490^*^), and to the C14:1c9 to C14:0 and C18:1c9 to C18:0 ratios (0.445^*^ and 0.462^*^, respectively). It was negatively correlated to the proportions of C5:0 (−0.407^*^), C6:0 (−0.458^*^), total SFA (−0.558^**^) and *de novo* FA (−0.441^*^). Two correlations were also found between milk FA profile and the nutritional value of the diet as calculated from the simulated bites: the crude protein content of the herbage was positively correlated to C18:3 n-6 proportion (0.531^*^) and the NDF content was negatively correlated to C12:1c9 proportion (−0.461^*^).

**Table 4:** Proportions of fatty acids (g/100 g total fatty acids) in milk fat distinguished by breed and period.

**Fatty acid**	**Breed**			**Period**		**SEM**	**Significance**		
	**Ho**	**Mo**	**Va**	**June**	**July**		**Breed**	**Period**	**Breed × period**
C4:0	3.44	3.53	3.24	3.50	3.31	0.100	ns	ns	ns
C6:0	1.94	1.93	1.81	2.00^a^	1.79^b^	0.050	ns	^*^	ns
C8:0	0.90	0.91	0.87	0.95^a^	0.83^b^	0.027	ns	^*^	ns
C10:0	1.69	1.64	1.59	1.77^a^	1.51^b^	0.059	ns	^**^	ns
C12:0	1.88	1.79	1.86	1.98^a^	1.76^b^	0.061	ns	^**^	ns
C12:1c9	0.048	0.051	0.052	0.050	0.051	0.002	ns	ns	ns
C14:0	8.21	8.37	8.05	8.62^a^	7.81^b^	0.221	ns	^**^	ns
C14:1c9	0.77	0.73	0.82	0.75	0.79	0.037	ns	^†^	ns
C15:0	1.28	1.28	1.35	1.22^b^	1.39^a^	0.040	ns	^**^	ns
C16:0	23.9	23.9	23.1	24.5^a^	22.7^b^	0.41	ns	^***^	^†^
C16:1c9	1.35	1.38	1.26	1.29	1.37	0.052	ns	ns	ns
C17:0	0.81	0.78	0.82	0.80	0.80	0.015	ns	ns	ns
C18:0	13.1	11.7	12.2	12.2	12.6	0.30	ns	ns	ns
C18:1t10	0.21	0.21	0.23	0.23	0.21	0.009	ns	ns	ns
C18:1t11	2.15	2.25	2.50	2.28	2.31	0.132	ns	ns	ns
C18:1c9	25.1	26.2	26.3	24.9^b^	26.8^a^	0.444	ns	^**^	ns
C18:2n-6	1.19	1.14	1.28	1.18	1.22	0.023	^†^	ns	ns
C18:3n-3	0.82^b^	0.87^ab^	0.97^a^	0.85	0.92	0.022	^*^	^†^	ns
C18:2c9t11	0.89	1.08	1.26	1.03	1.129	0.056	ns	ns	ns
ECFA	55.6	54.2	53.1	55.9^a^	53.0^b^	0.61	ns	^**^	ns
OCFA	2.89	2.87	3.01	2.79^b^	3.05^a^	0.060	ns	^***^	ns
BCFA	3.01	3.00	3.08	2.99	3.08	0.076	ns	ns	ns
SFA	60.9	59.6	58.6	61.1^b^	58.2^a^	0.54	ns	^**^	ns
MUFA	33.7	35.0	35.3	33.5^b^	35.9^a^	0.47	ns	^**^	ns
PUFA	4.53	4.62	4.16	4.56^b^	4.97^a^	0.124	ns	^*^	ns
Σcis18:1	26.4	27.5	27.6	26.1^b^	28.2^a^	0.46	ns	^**^	ns
Σtrans18:1	3.46	3.60	3.96	3.60	3.75	0.168	ns	ns	ns
Σn-6	1.52	1.39	1.55	1.46	1.51	0.029	^†^	ns	ns
Σn-3	1.12	1.12	1.25	1.12^b^	1.21^a^	0.027	ns	^*^	ns
**Ratios**									
n-6 to n-3	1.37^a^	1.24^b^	1.25^b^	1.31^a^	1.26^b^	0.019	^*^	^*^	ns
C14:1c9 to C14:0	0.094	0.086	0.102	0.087^b^	0.102^a^	0.004	ns	^**^	ns
C18:1c9 to C16:0	1.05	1.12	1.15	1.02^b^	1.19^a^	0.034	ns	^***^	ns
Σ *de novo* synthesized fatty acids	18.0	18.3	17.4	18.8^a^	17.0^b^	0.42	ns	^*^	ns

Significance levels:

*** p ≤ 0.001,

** p ≤ 0.01,

* p ≤ 0.05,

† p ≤ 0.10, ns p > 0.10.

a, b *Least square means with different superscript letters significantly differ at p < 0.05*.

*BCFA, branched-chained fatty acids; ECFA, even-chained fatty acids; Ho, Holstein; Mo, Montbéliarde; MUFA, mono-unsaturated fatty acids; OCFA, odd-chained fatty acids; PUFA, polyunsaturated fatty acids; SFA, saturated fatty acids; Va, Valdostana Red Pied*.

### Prevalence of Milk Terpenes and Their Relation With Grazing Behavior

A total of 16 different terpenes were identified in the milk of the three breeds, whereof the 11 most frequently found terpenes are presented in Table 5. From these 16 terpenes, α- and β-pinene, camphene, γ-terpinene, α- and β-caryophyllene were found in all 24 samples. Sabinene was detected in 22 samples out of 24, limonene in 21, β-myrcene and terpinolene in 19, and α-phellandrene in 16. The other compounds occasionally detected were α-thujene, δ3-carene, ocimene and linalool. Regardless of breed or period, the three most abundant terpenes found in milk were limonene, β-caryophyllene and γ-terpinene, with in average 24.6, 23.8, and 10.2 %, respectively. Milk terpenes did not vary among breeds, but a few differences occurred between the two experimental periods. This was Specifically an increase in camphene (+2.98 %) and α-caryophyllene (+2.64 %) prevalence in July compared to June. Terpinolene and β-caryophyllene tended to increase in July, too (+1.50 and +9.18%, *p* = 0.096 and 0.088, respectively), while β-myrcene tended to decrease (−1.53%, *p* = 0.068). The milk terpenes were not correlated to the proportions of different botanical groups in the bites of the cows, except for α-caryophyllene that was negatively correlated with the estimated grasses intake (−0.475^*^). It was also negatively correlated to the ADF content of the bites (−0.454^*^).

**Table 5:** Prevalence of the most abundant terpenes in milk (proportion of individual peak area over total peak area, %) distinguished by breed and period.

**Terpene**	**Breed**			**Period**		**SEM**	**Significance**		
	**Ho**	**Mo**	**Va**	**June**	**July**		**Breed**	**Period**	**Breed × period**
									
Limonene	26.7	30.1	17.0	29.4	19.8	3.96	ns	ns	ns
β-Caryophyllene	21.2	23.5	26.8	19.2	28.4	2.73	ns	^†^	ns
γ-Terpinene	10.56	12.15	7.96	8.98	11.47	1.323	ns	ns	ns
α-Pinene	8.75	9.61	10.72	10.03	9.36	0.599	ns	ns	ns
β-Pinene	8.71	8.80	10.85	7.90	11.01	0.891	ns	ns	ns
α-Caryophyllene	3.49	3.15	5.03	2.56^b^	5.21^a^	0.602	ns	^*^	ns
Camphene	3.37	3.43	4.55	2.29^b^	5.27^a^	0.639	ns	^*^	ns
β-Myrcene	2.30	2.82	2.24	3.22	1.69	0.363	ns	^†^	ns
Terpinolene	2.00	1.74	2.83	1.44	2.94	0.394	ns	^†^	ns
Sabinene	1.29	1.43	1.96	1.76	1.36	0.170	ns	ns	ns
α-Phellandrene	0.32	0.61	0.63	0.30	0.74	0.129	ns	ns	ns

Significance levels:

* p ≤ 0.05,

† *p ≤ 0.10, ns p > 0.10*.

a, b *Least square means with different superscript letters significantly differ at p < 0.05*.

*Ho, Holstein; Mo, Montbéliarde; Va, Valdostana Red Pied*.

## Discussion

### Differences Among Breeds in Grazing Selection

In continuous grazing conditions on a biodiverse pasture, according to the IS, cows of the least specialized breed (Va) were less selective on pasture than those of the highly specialized breed (Ho). This was particularly the case with mature herbage. Grazing behavior of Mo seems to be more similar to that of Ho, even though they were a little less selective. However, these differences in grazing selection were minimal compared to those expressed within breeds on pastures with different botanical composition or under different grazing management (21, 27), as was also observed in the present study, when forage of different vegetation growth stage was offered in June and July. Still, the breed differences in grazing selection were lower than expected and were in contrast to the results of Farruggia et al. (35), who showed that lactating cattle with high nutrient requirements grazed more selectively than dry cows, and Pauler et al. (22, 23), who found that more productive Angus × Holstein cattle grazed more selectively than Highland cattle. However, Dumont et al. (36) also did not find relevant differences in grazing behavior among traditional and specialized breeds. The few differences among cow breeds highlighted by the IS did not lead to a significant difference in composition and estimated digestibility of the simulated bites in our experiment. This indicates that, even though cows selected or avoided some species while grazing, the corresponding changes in DM quantities found in the simulated bites remained too low to make a clear difference in their proportion of the total daily diet. Concerning season and evolution of the herbage, cows avoided forbs and mature vegetation less in June than in July, probably because they already overgrazed the preferred patches with grasses during the beginning of the grazing cycle (24, 37, 38). In July, they selected the vegetative regrowth and avoided legumes less. This can be explained by the observation that the latter botanical group regrows rapidly (21) and therefore was more accessible at that time than at the end of the first grazing cycle. These changes in grazing selection and the increased small proportion of legumes are coherent with the higher crude protein content in the simulated bites, together with their lower ADF content, in July compared to June. Finally, cows seem to have adapted their grazing behavior to what was offered during the respective season, regardless of breed. Further studies would be required to strengthen our findings over a larger number of animals.

### Differences Among Breeds in Milk FA Profile

The differences among cow breeds in milk yield and gross composition were previously discussed in Koczura et al. (24). Briefly, the lower milk fat content in Va milk compared to Mo is typical of Va breed (39, 40), as this dual-purpose breed is not specifically selected for a high milk fat content. The lower fat content of Ho compared to Mo is in accordance with previous results comparing Ho and Mo reared in the same conditions (41). The lower fat and protein yields of Ho compared to Mo could be due to the lower adaptability of Ho breed to cover their high requirements without concentrate supplements, resulting in a negative energy balance, and lower fat and protein yield (24). Regarding FA composition of milk fat, several studies already investigated the link between the composition of diverse pastures and milk quality [e.g., (16, 18, 42)], but only a few allowed to link the selective behavior that ruminants exhibit on pasture with their milk composition (27, 38). These authors illustrated how the ability of an animal to feed on forbs instead of grasses has an effect on the ruminal microbial population, with PSC partially inhibiting the biohydrogenation of dietary PUFA, entirely or at certain steps. This results in milk richer in these FA, and particularly in n-3 FA (43). However, this effect is especially visible when comparing selection on highly biodiverse pastures with grasslands with very different proportions of grasses and forbs (18). In the present experiment, all cows grazed on the same biodiverse pasture. The results, therefore, suggest that, even though some preferences were expressed, the differences in the material eaten among breeds remained too low to affect the milk FA profile. This is consistent with the similar FA profile of the milk found for the three breeds, especially in terms of MUFA, PUFA, and CLA proportions of the milk fat. These groups of FA were actually correlated to the herbage evolution over the season and, more specifically, to the proportion of dry material in the simulated bites, regardless of breed. The cause for that is the advanced herbage phenological stage in June compared to July measurement period, which is known to increase MUFA and decrease PUFA and CLA proportions of the milk fat (18, 42). Only one particular FA, the major n-3 FA C18:3 n-3, was found to be always higher in Va milk fat compared to Ho milk fat, without any correlation to diet selection or period. This FA is a substrate for ruminal biohydrogenation, leading to the production of C18:1t11, ultimately being saturated to C18:0 (43). Although not significant, C18:1t11 was also numerically higher in Va than Ho milk fat. This suggests that C18:3 n-3 is less biohydrogenated in the rumen of Va than Ho cows, and this could probably be explained by breed differences in the rumen microbial population and metabolism rather than grazing behavior. This aspect should be further investigated, especially on a larger number of animals.

### Differences Among Breeds in the Prevalence of Milk Terpenes

To our knowledge, this is the first study directly linking diet selection of dairy cows and the occurrence of terpenes in their milk. Terpenes are absorbed directly from the diet (9), and most of them are transferred directly into milk and are subject to no or minor changes (44, 45). Some authors demonstrated that terpenes can also be partially biohydrogenated and isomerized by the rumen microbial population, leading to additional terpenes in milk (46). In the present experiment, the investigated pasture was rich in forbs, such as plants from the *Apiaceae* family and *Thymus serpyllum*, both containing pinenes and caryophyllenes (47, 48). The presence of this kind of compounds in the milk of the experimental cows suggests that pinenes and caryophyllenes indeed originated from the diet. Several previous studies already identified β-caryophyllene in the milk of grazing cows (10, 11). However, unlike our hypothesis, it seems difficult to directly relate terpene prevalence in milk and grazing behavior of the cows. Accordingly, in the present study no significant correlations were found between terpene prevalence and the proportion of the different botanical groups in the simulated bites. This could be partially due to the limited number of animals in our experiment. However, if a biological link was underlying between grazing selection and milk composition, significant correlations would have been expected even with few animals, probably with a poor correlation coefficient that would have been improved by increasing the number of animals, but it was not the case. This suggests that the selection of a single species or family could be responsible for the transfer of such molecules (5), rather than the overall proportion of forbs or mature vegetation in the sward. Besides, Lejonklev et al. (49) showed that terpenes from essential oils can be transferred to milk by both ingestion and inhalation. Cows may therefore also have taken in terpenes while breathing, which would explain why the latter are not correlated to the simulated bites. The few differences that occurred in milk terpene prevalence were related to the grazing period: the vegetative stage of the pasture evolved after the first grazing cycle and most probably led to a different terpene composition of the herbage (5, 50). Indeed, a parallel increase was observed by Tornambé et al. (10) between milk terpenes concentration and the variation in the phenological stage of the herbage. Even though concentrations of terpenes found in milk are low, it would be interesting to manufacture cheeses with the milk of the three breeds and investigate the link between terpene profile, microbial development and potential further influences on cheese sensory properties. Such an effect could be expected because some terpenes have been found to have antimicrobial effects. Rivas da Silva et al. (47) for instance demonstrated that positive enantiomers of α- and β-pinene used in synergy (250 μg/mL) can have a bactericidal effect against methicillin-resistant *Staphylococcus aureus*.

### Implications

The overall small differences among the autochthonous, more robust Va, the intermediate Mo and the Ho highly specialized for milk production may suggest that in low-input mountain grazing systems, the individual animal's adaptability in the short term could actually be more important than its breed. It has to be mentioned, though, that all cows were in their late lactation and therefore even the Ho were not in a situation of high nutrient requirements. Breed differences could be clearer in other stages of lactation. Breed also seems to have less influence on milk quality than herbage composition. Other criteria than grazing behavior should be investigated in order to assess the role of autochthonous breeds in the multi-performance of future low-input grazing systems. Indeed, individual adaptation in the short term may result in different long-term breed responses, i.e., in reproduction or productive lifespan. Further investigation on a larger number of animals would reinforce our findings.

## Data Availability Statement

The raw data supporting the conclusions of this article will be made available by the authors, without undue reservation.

## Ethics Statement

No ethical approval was required because no invasive samples have been taken on the animals and all procedures carried out followed the Certificate of Authorization to Experiment on Living Animals No. D 15-114-01 delivered by the French government to Marcenat INRAE experimental facility.

## Author Contributions

MKo, MC, BM, MKr, and JB contributed to the conception and design of the study. MKo, MB, and GT organized the experiment. MKo supervised the experiment. MKo, MB, and BM participated in behavioral observation and milk sampling. MC performed the fatty acids analysis. MMu and MMa performed the terpene analysis. MKo and MC processed the data and equally contributed to the writing of the manuscript. MC performed the statistical analysis. MKr and JB supervised MKo in the frame of her doctoral thesis. All the authors contributed to manuscript revision, and all read and approved the submitted version.

## Conflict of Interest

The authors declare that the research was conducted in the absence of any commercial or financial relationships that could be construed as a potential conflict of interest.
